# Factors Associated with Rural Residents’ Contract Behavior with Village Doctors in Three Counties: A Cross-Sectional Study from China

**DOI:** 10.3390/ijerph17238969

**Published:** 2020-12-02

**Authors:** Linni Gu, Rui Zhu, Zhen Li, Shengfa Zhang, Jing Li, Donghua Tian, Zhijun Sun

**Affiliations:** 1 Business School, Beijing Normal University, 19 Xinjiekou Wai Street, Haidian District, Beijing 100875, China; smartgu@163.com (L.G.); zhenlii2018@163.com (Z.L.); 2 China Academy of Social Management/School of Sociology, Beijing Normal University, 19 Xinjiekou Wai Street, Haidian District, Beijing 100875, China; zhurui@bnu.edu.cn; 3 School of Social Development and Public Policy, Beijing Normal University, 19 Xinjiekou Wai Street, Haidian District, Beijing 100875, China; zhangshengfa1988@sina.com (S.Z.); 15910518836@163.com (J.L.); tian65216@hotmail.com (D.T.)

**Keywords:** village doctor contract service, NRCMI, trust, reimbursement rate, drug treatment effect, family doctor, rural area

## Abstract

Historically, cooperative medical insurance and village doctors are considered two powerful factors in protecting rural residents’ health. However, with the central government of China’s implementation of new economic policies in the 1980s, cooperative medical insurance collapsed and rural residents fell into poverty because of sickness. In 2009, the New Rural Cooperative Medical Insurance (NRCMI) was implemented to provide healthcare for rural residents. Moreover, the National Basic Drug System was implemented in the same year to protect rural residents’ right to basic drugs. In 2013, a village doctor contract service was implemented after the publication of the *Guidance on Pilot Contract Services for Rural Doctors*. This contract service aimed to retain patients in rural primary healthcare systems and change private practice village doctors into general practitioners (GPs) under government management. Objectives: This study investigates the factors associated with rural residents’ contract behavior toward village doctors. Further, we explore the relationships between trust, NRCMI reimbursement rate, and drug treatment effect. We used a qualitative approach, and twenty-five village clinics were chosen from three counties as our study sites using a random sampling method. A total of 625 villagers participated in the investigation. Descriptive analysis, chi-squared test, *t*-test, and hierarchical logistic analyses were used to analyze the data. Results: The chi-squared test showed no significant difference in demographic characteristics, and the *t*-test showed a significant difference between signed and unsigned contract services. The results of the hierarchical logistic analysis showed that trust significantly influenced patients’ willingness to contract services, and the drug treatment effect and NRCMI reimbursement rate moderated the influence of trust. Conclusion: Our findings suggest that the government should aim to strengthen trust in the doctor–patient relationship in rural areas and increase the NRCMI reimbursement rate. Moreover, health officers should perfect the contract service package by offering tailored contract services or expanding service packages.

## 1. Introduction

In rural China, village clinics have always served as the primary healthcare organization in the three-tiered rural public health system, with village doctors acting as rural residents’ first point of contact with the healthcare system [1]. In the 1950s, the health status of China’s population was notoriously poor, with an average life expectancy of approximately 40 years [2]. To solve this problem, the central government cultivated “barefoot doctors”—who became village doctors after the 1980s—and implemented the Cooperative Medical Scheme (CMS), which was the predecessor to the New Rural Cooperative Medical Insurance (NRCMI). The CMS was welcomed by rural residents and highly supported by the collective organization at that time. Its funding was supplied by collective welfare funds as well as payments from the collective organization [3]. Each member of the collective organization would pay only one Yuan per year and five Fen (0.05 Yuan) for each visit to the clinic. By the end of 1958, it was estimated that almost one-fifth of the collective organizations in the country had established CMS [4,5]. However, with the central government of China implementing new economic policies, the rural health delivery system was seriously altered. Village clinics were largely privatized due to the disintegration of the commune and the collapse of the collective economy. More than 50% of rural village clinics were run by individuals. The fee-for-service model was dominant, and fees were mostly charged for dispensing drugs. Many rural residents could not access the necessary healthcare because of their poor financial situation. Many rural residents, therefore, became poor due to sickness. The NRCMI was fully implemented in 2009 as part of a new round of health reform. Likewise, the National Development and Reform Commission and the National Health Commission of the People’s Republic of China, together with nine other departments, jointly launched the *National Basic Drug System*, which proposed that primary medical and healthcare institutions must use basic drugs to treat regular illness. The drugs listed in the basic drug list are covered by NRCMI.

In rural China, residents do not like to visit doctors in village clinics due to poor healthcare service. To solve this problem, the National Health Commission of the People’s Republic of China published the *Guidance on Pilot Contract Services for Rural Doctors* in 2013, aimed at providing comprehensive, coordinated, and preventative public healthcare to all villagers. The contract service is similar to family medicine in developed countries, and it encourages village doctors to provide demand-tailored public health services, similar to GPs. In 2018, the State Council issued the *Opinion on Reforming and Improving the Training and Employment Incentive Mechanism of General Practitioners* [6], which proposed encouraging village doctors who had acquired a certification of practice as assistant doctors to participate in training as GPs and pointed out that by 2030, 10,000 inhabitants will have to be serviced by one GP in rural areas. The team of contract services in rural areas consist of GPs (village doctors trained as GPs; in this study, village doctors are replaced with GPs), public health physicians, nurses, and village doctors. They provide basic health services, public health, tracking services for the target population, and a referral service.

However, the contract service was not implemented smoothly in all rural areas. Previous studies have found that existing contract services were superficial and deceptive, pursued only quantity, were not accepted by patients, and lacked guarantee [7,8]. The contract rate was low, under 30%, in rural areas [9]. To solve these problems and improve the implementation of family doctor contract services, numerous studies have explored the factors that influence patients’ willingness to utilize the contract services. A previous study found that the lack of sufficient incentive for doctors was the major reason for inefficient contract services [8]. A similar study discovered that the absence of a third-party assessment mechanism led to ineffective contract service [10], while yet another found that doctors’ competence and doctors’ attitudes were the main reasons for the low contracting rate [11]. Other studies also found that patients’ satisfaction with service quality was the main influencing factor of patients’ acceptance of the family doctor contract service [12].

Based on the above background and literature analysis, this study will explore the factors that influence patients’ willingness to use contract services in rural areas. The following factors will be investigated: patients’ trust in doctors, satisfaction with the NRCMI policy, drug utilization, service quality of doctors, and demographic variables. The main research question is, what are the significant factors associated with villagers’ willingness to use village doctor contract services in rural areas after controlling for demographic characteristics?

## 2. Materials and Methods

### 2.1. Study Setting and Sample

This study was conducted in three counties—Dafeng district, Yinan county, and Wufeng county—of rural China. The counties were chosen according to their economic status, geographic situations, investigation feasibility, and financial support. DF district is located in the eastern plain of Jiangsu province and reported a GDP of 647.48 billion Yuan in 2017 [13]; YN county is located west of Shandong Province, with a GDP of 262 billion Yuan in 2017 [14]; WF is a minority county and is located southeast of Hubei Province, with a GDP of 65.49 billion Yuan in 2017 [15]. The three counties represent not only different economic status and locations but also show significant differences in geography; specifically, the geological feature of DF county is plains, YN presents an upland feature, and WF is a mountain area. DF was the first district to implement the village doctor contract service, with WF and YN implementing the service policy after DF. Considering the contract service, DF provides a specialized contract service to villagers, while the other two counties do not.

Using a simple random sampling method, 25 village clinics were selected from the town’s village clinics list provided by local officers at the town’s central hospitals in the three areas. Participants were selected according to daily outpatient visits over one year and the contract service list in each village clinic. Considering the demographic representation, the sample size was calculated using the following formula [16,17,18]:

(1) $$n=\frac{t^{2}\times p\left(1-p\right)}{e^{2}}$$

where the confidence level is 0.05 (two sides), *t* = 1.96, *p* = 30% (according to a previous study) [18,19], and the allowed error is *e* = 0.04. The calculation showed that approximately 504 participants were a sufficient sample size. Considering the facilitation of the sample size’s proportional allocation, the sample size was expanded into an integer of 600, with each county allocated 200 participants. The nonresponse rate was controlled at 10%, and the actual sample was set at 625 participants. However, we also considered that the number of rural residents had declined due to urbanization. The inclusion criteria for participants were (1) having attended the village clinics during the last year, (2) ≥18 years old, and (3) good hearing, speaking, and mental health. Ultimately, 574 valid questionnaires were included after 51 questionnaires were excluded from the original 625 samples.

### 2.2. Data Collection

Based on a literature review and expert guidance, a self-designed questionnaire was used for data collection. The questionnaire included four parts: (1) demographic characteristics, (2) health policy variables and drug utilization, (3) health service quality measurement, and (4) trust measurements. Data were collected from October to December 2017 in 25 village clinics in the three counties. Voluntary investigators assisted with completing the questionnaires. All investigators were trained to fully understand the questionnaire and had the skills for face-to-face interviews. All participants were enrolled in the clinic’s meeting room and doctor’s offices to complete the structured face-to-face questionnaire interview. Local village doctors helped us to understand the local dialects when patients could not speak Mandarin. The time to complete each questionnaire ranged from 30 to 45 min, depending upon different participants’ situations. All participants received a towel as a token of appreciation after completing the questionnaire. Qualified supervisors checked all completed questionnaires after the interviews were completed.

### 2.3. Ethical Considerations

This study was approved by the Ethics Committee of the School of Social Development and Public Policy at Beijing Normal University (project identification code: 2017YFB2101100). The investigation was voluntary; all participants were free to refuse to participate, and written informed consent was obtained. All personal information was kept confidential, and reporting was made anonymous.

### 2.4. Measures

#### 2.4.1. Participants’ Characteristics

Demographic information and economic variables were measured, including gender, age, educational status, and family income.

#### 2.4.2. Drug Utilization

Drug utilization was measured as a polytomous variable. It included drug price and drug treatment effects. The questions asked were, “what do you think of the drug’s price?” and “what do you think of the drug’s treatment effect?”. The responses were “low, appropriate, and high” and “poor, moderate, and good”, respectively.

#### 2.4.3. Contract Service

Contract service was measured with the following question: “Did you sign up for the contract service with the village doctor?” Answers were dichotomized as “no = 0” and “yes = 1”.

#### 2.4.4. Service Quality

The Chinese version of the ServQual scale was used to measure village doctors’ service quality. It consists of 22 items and measures five dimensions: tangibility, reliability, responsiveness, assurance, and empathy. The responses ranged from 1 = very bad to 9 = very good. The reliability of the scale was confirmed in a previous study with a Cronbach’s α of 0.94 [20].

#### 2.4.5. Patient Trust

The translated Chinese version of the Wake Forest Physician Trust Scale was used to measure patients’ trust in doctors. This scale is a widely-used assessment tool in China and consists of ten items measuring two dimensions of patients’ trust in doctors. Responses were scored using a 5-point Likert scale. The reliability of the scale was confirmed in a previous study (Cronbach’s α = 0.89) [21].

#### 2.4.6. Medical Insurance Policy

The NRCMI was measured with one question on the NRCMI’s reimbursement rate: “What do you think of the NRCMI’s reimbursement rate?” (low, suitable, and high).

### 2.5. Statistical Analysis

To investigate whether health service quality and other variables can influence patients’ willingness to contract with a village doctor, the following statistical analyses were applied: first, a descriptive analysis was conducted to analyze the demographic characteristics of participants, and the means for continuous variables and frequencies for categorical variables were calculated. Second, Pearson’s chi-squared test and the *t*-test were used to compare sociodemographic characteristics, drug utilization, policy NRCMI, service quality, and trust between signed and nonsigned contract services. Third, a hierarchical logistic regression analysis was employed to explore health service quality and other variables that influence patients’ willingness to contract with village doctors. In the first step, demographic characteristics and service quality were entered in the model; second, policy NRCMI was added; next, drug utilization was entered in Model 3, and lastly, patient trust was added to the model. All the data were analyzed using Stata Software (version SE15, Stata Corp., College Station, TX, USA); *p*-values < 0.1 were considered to be statistically significant.

## 3. Results

### 3.1. Correlation between Contract Service and Drug Utilization, NRCMI, Trust, Service Quality, and Demographic Variables

As shown in Table 1, the correlation between contract service and drug utilization, NRCMI, trust, service quality, and demographic characteristics were analyzed using a pairwise Pearson’s correlation. Contract service was highly correlated with drug treatment effect (*p* ≤ 0.01), reimbursement rate of NRCMI (*p* ≤ 0.01), patient trust in doctors (*p* ≤ 0.01), and doctors’ health service quality (*p* ≤ 0.01).

### 3.2. Trust Significantly Influenced Contract Service 

Table 2 summarizes the results of the hierarchical logistic regression for factors that influenced patients’ acceptance of contract services. The first model showed that service quality significantly influenced patients’ willingness to sign up for a contract service after controlling for confounders; this result is consistent with previous studies. Model 2 showed that the influence of service quality decreased with the NRCMI reimbursement rate added to the model. Meanwhile, the results showed that a high reimbursement rate significantly influenced patients’ willingness to contract services; patients who considered reimbursement rates to be high were more likely to contract with village doctors. Model 3 showed that the influence of service quality further declined when drug utilization (drug treatment effect and drug price) was added to the model. Good drug treatment significantly influenced patients’ willingness to contract services. Likewise, patients who considered drug treatments to be effective were likely to contract with village doctors. Model 4 showed that the influence of service quality disappeared when trust was added and that trust had a significant influence on patients’ willingness to contract service.

### 3.3. Patient Trust Was Moderated by Drug Treatment Effect and Reimbursement Rate of NRCMI

**Figure 1:**
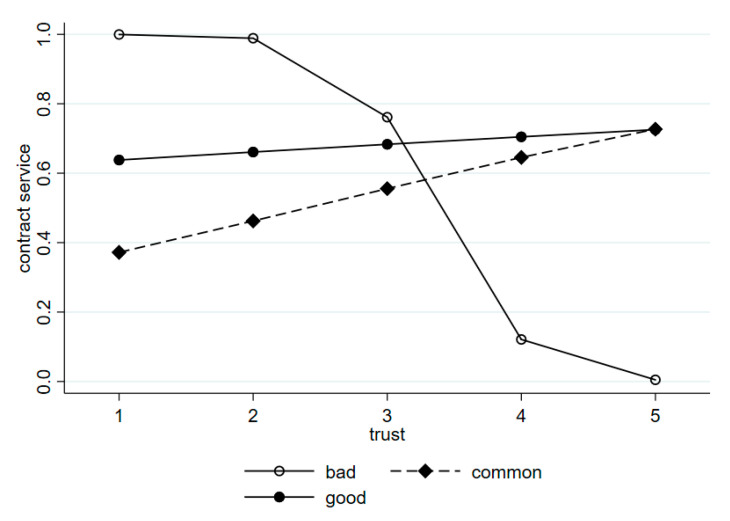
Relationship between trust and drug treatment effect on patient willingness to contract.

**Figure 2:**
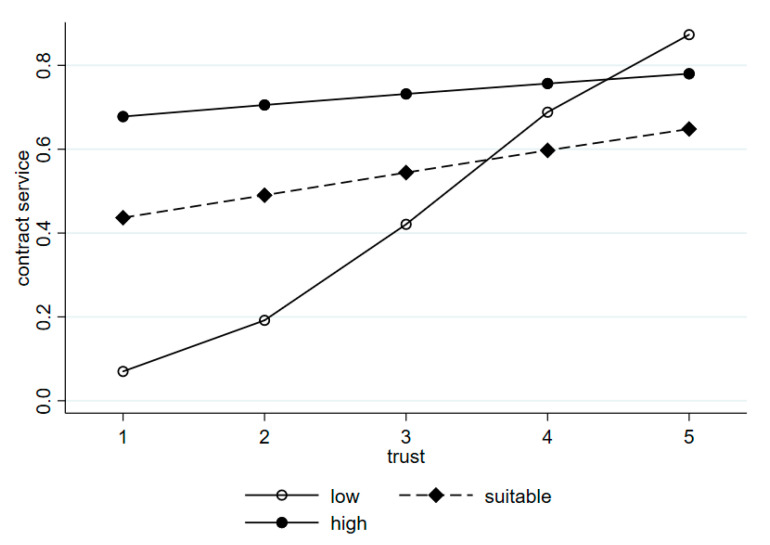
Relationship between trust and reimbursement rate of NRCMI on patient willingness to contract.

The interaction terms (reimbursement rate × trust and drug treatment effect × trust) were also examined in Model 5. The results showed that patient trust was moderated by the patient perception of drug treatment effect and NRCMI reimbursement rate. The influence of trust on patients’ willingness to contract was significant (*p* < 0.1) among patients who considered the drug treatment effect moderate and good. Likewise, the influence was significant (*p* < 0.1) among patients who considered the reimbursement rate high. Figure 1 and Figure 2 show the relationship between patient trust and patient perception of drug utilization and NRCMI on patient willingness to contract. Among the patients who considered the drug treatment effect moderate and good, their willingness to contract would increase with the increase in trust in doctors, compared with patients who considered the drug treatment effect to be bad (β = 2.151, β = 2.158; Figure 1). Considering the patients who reported the reimbursement rate to be high, their willingness to sign contracts slowly decreases as trust increases to a certain level, compared with patients who considered the reimbursement rate to be low (β = −1.279; Figure 2).

## 4. Discussion

This study examines the factors that influence patients’ willingness to contract the services of village doctors using hierarchical regression. An interesting finding is that patient trust plays an important role in patients’ willingness to sign up for a contract service. We also found that the NRCMI reimbursement rate and drug treatment effect significantly influenced patients’ willingness to contract services. We also investigated the interaction terms (reimbursement rate × trust and drug treatment effect × trust) in this study.

It is well known that health service quality is associated with the retention of current customers and the attraction of new ones, decreased costs, and enhanced hospital image [22,23]. Previous studies showed that service quality was the most important factor influencing patients’ decision to contract with family doctors [24,25,26]. However, the current study’s results show that service quality has no significant effect on patients’ willingness to contract with village doctors. A possible reason for the different results may be the different study subjects. Previous studies chose urban patients as their study subjects, while this study focused on rural patients. The contrary result illustrates that patients in rural and urban areas have different requirements for healthcare services and doctor–patient relationships. Comparatively speaking, urban patients attach greater importance to health service quality than rural patients do. The deeper reason may be the different interpersonal relationships in the urban–rural dual structure. According to sociologist Anthony Giddens’ structure theory [27], interpersonal relationships in urban areas are often with strangers, while most interpersonal relationships are with acquaintances in rural areas. This means that patients in urban areas placed a higher value on doctors’ service quality than rural patients.

We also found that the NRCMI reimbursement rate, drug treatment effect, and patients’ trust significantly influence patients’ willingness to contract services in rural areas. Patients who considered reimbursement rates to be high were more likely to sign contract services with village doctors. This is because a contracted service provides a higher reimbursement rate and broader reimbursement items. According to contract service policy, medical insurance should provide support for the implementation of contract services, including service fees for contracts, expanding the scope of reimbursement, declining the reimbursement’s starting line, and increasing illness items and drug types in reimbursement. Specifically, contracted patients would be reimbursed more than those who are noncontracted when they suffer from the same illness [24]. It is easy to understand that drug treatment effects influence patients’ willingness to contract services. If a patient’s perception of drug treatment effect is good, that patient’s willingness to contract will increase. Contrarily, if a patient’s perception of drug treatment effect is bad, they will not sign contracts with village doctors. Lastly, trust is the most important factor that influences patients’ willingness to contract services, rather than health service quality, in rural areas. Numerous studies have illustrated that trust plays a critical role in the doctor–patient relationship [28,29,30]. This is because of the acquainted interpersonal relationships in rural areas, mentioned above, which means that patients and doctors are often quite familiar with each other in the villages. Regarding the background of village doctors, most of them are local people. They are familiar with their patients’ illness, family health history, and economic status. Thus, the trust relationships between them are easily established. Furthermore, according to social trust theory, trust plays a pivotal role in an acquaintance society [31,32]. Thus, it is easy to understand that patients in rural areas contracted with village doctors depending on their trust relationship.

The most important finding in this study is that the relationship between patients’ signing up for contract service and patient trust is moderated by the reimbursement rate and drug treatment effect. In other words, the reimbursement rate and drug treatment effect weaken patients’ trust in contracting with village doctors. This illustrates that patients who are contracted with village doctors not only think about their trust relationship but also consider medical expenditures and drug utilization. Historically, rural residents of China in the 1980s to 1990s were not covered by medical insurance. At that time, numerous rural residents fell into poverty due to illness, and some fell into severe poverty because of catastrophic illness. A recent study pointed out that millions of people are impoverished because of health expenditure or catastrophic amounts of money spent on healthcare [33]. Thus, medical insurance plays a pivotal role in protecting residents from poverty. From 2009, primary health care institutions are required to use drugs from the basic drug list, which are covered by the NRCMI. Rural residents who consult doctors in village clinics will also get a higher NRCMI reimbursement rate. Even so, many residents prefer higher-level hospitals when seeking medical help.

The limitations of this study should be acknowledged. First, the study sites were only from East and Central China, meaning that the western provinces were not selected in this study; thus, the results cannot be extrapolated to the whole of China. Second, although certain confounders were controlled in the study, some other confounders may have been overlooked, which should be considered in future research. Third, this study only explored the factors that influence patients’ willingness to sign up for a contract service; however, the mechanism that influences patients’ contracting is unknown. Future studies should investigate this mechanism.

## 5. Conclusions

This study empirically investigated factors that influenced rural residents’ willingness to sign up for contract services with family doctors in rural areas. Trust was shown to play a pivotal role in rural residents signing up for contract services with village doctors. However, the relationship between trust and rural residents’ willingness to contract with village doctors were moderated by medical insurance reimbursement rates and drug treatment effects. These findings suggest that the government should formulate policies to strengthen the doctor–patient trust relationship in rural areas to enhance the rate of contract service and to retain more patients in primary health. Moreover, the government should provide NRCMI to support contract services. Meanwhile, local health officers should perfect the contract service package by offering tailored contract services or expanding service packages. In this way, overcrowding in large or popular hospitals would be relieved and the utilization of the primary healthcare system would be enhanced.

## Figures and Tables

**Table 1:** Pairwise correlation between contract service and drug utilization, New Rural Cooperative Medical Insurance (NRCMI), trust, service quality, and demographic variables.

Variables	Contract Service	Drug Treatment Effect	Drug Price	Reimbursement Rate	Reimbursement Procedure	Trust	Service Quality	Age	Education	Family Income
Contract service	1									
Drug treatment effect	0.20 ***	1								
Drug price	−0.12 *	−0.26 ***	1							
Reimbursement rate	0.19 ***	0.21 ***	−0.24 ***	1						
Reimbursement procedure	−0.07	−0.16 ***	0.26 ***	−0.25 ***	1					
Trust	0.26 ***	0.30 ***	−0.34 ***	0.19 ***	−0.09	1				
Service quality	0.19 ***	0.39 ***	−0.48 ***	0.24 ***	−0.22 ***	0.58 ***	1			
Age	0.03							1		
Education	0.04							−0.25 ***	1	
Family income	0.02							−0.28 ***	0.21	1

* *p* < 0.1; *** *p* < 0.01.

**Table 2:** Hierarchical multivariate logistic regression of the influence factors associated with contract service.

	Variables	β	SE	*p*-Value	(95% CI)	OR
**Model 1**	**Sex**					
	Male vs. female	−0.362	0.187	0.053	(−0.730, 0.005)	0.696
	**Age**					
	41–59 vs. ≤40	−0.385	0.223	0.084	(−0.822, 0.052)	0.681
	≥60 vs. ≤40	−0.091	0.281	0.745	(−0.642, 0.459)	0.913
	**Education**					
	Middle school vs. primary school or lower	0.134	0.424	0.751	(−0.697, 0.966)	1.144
	High school or higher vs. primary school or lower	0.281	0.466	0.547	(−0.633, 1.195)	1.324
	**Income**					
	10,000–29,999 vs. ≤9999	−0.429	0.219	0.051	−0.858, 0.001	0.651
	≥30,000 vs. ≤9999	0.191	0.273	0.484	−0.344, 0.726	1.211
	**Service quality**	0.471	0.100	<0.001	0.274, 0.668	1.601
	Pseudo R^2^	0.053				
	Log likelihood	−359.035				
	Chi-squared	25.357				
	Akaike crit. (AIC)	721.866				
	Bayesian crit. (BIC)	747.780				
	*N*	574				
**Model 2**	**Sex**					
	Male vs. female	−0.422	0.193	0.029	−0.801, −0.042	0.656
	**Age**					
	41–59 vs. ≤40	−0.405	0.228	0.075	−0.851, 0.041	0.667
	≥60 vs. ≤40	−0.225	0.291	0.440	−0.796, 0.346	0.799
	**Education**					
	Middle school vs. primary school or lower	0.098	0.440	0.824	−0.765, 0.962	1.103
	High school or higher vs. primary school or lower	0.193	0.481	0.688	−0.750, 1.137	1.213
	**Income**					
	10,000–29,999 vs. ≤9999	−0.241	0.230	0.294	−0.692, 0.209	0.785
	≥30,000 vs. ≤9999	0.326	0.284	0.251	−0.230, 0.883	1.386
	**Reimbursement rate of NRCMI**					
	Suitable vs. low	−0.056	0.363	0.877	−0.768, 0.656	0.945
	High vs. low	0.839	0.385	0.029	0.084, 1.594	2.314
	**Service quality**	0.283	0.109	0.009	0.070, 0.497	1.328
	Pseudo R^2^	0.087				
	Log likelihood	−346.227				
	Chi-squared	38.262				
	Akaike crit. (AIC)	712.015				
	Bayesian crit. (BIC)	746.552				
	*N*	574				
**Model 3**	**Sex**					
	Male vs. female	−0.441	0.196	0.025	−0.826, −0.057	0.643
	**Age**					
	41–59 vs. ≤40	−0.404	0.230	0.080	−0.856, 0.048	0.668
	≥60 vs. ≤40	−0.197	0.294	0.502	−0.773, 0.379	0.821
	**Education**					
	Middle school vs. primary school or lower	0.073	0.445	0.870	−0.799, 0.944	1.076
	High school or higher vs. primary school or lower	0.160	0.485	0.741	−0.791, 1.112	1.174
	**Income**					
	10,000–29,999 vs. ≤9999	−0.219	0.233	0.349	−0.676, 0.239	0.804
	≥30,000 vs. ≤9999	0.316	0.288	0.274	−0.205, 0.881	1.371
	**Reimbursement rate of NRCMI**					
	Suitable vs. low	−0.096	0.366	0.794	−0.813, 0.622	0.909
	High vs. low	0.757	0.391	0.053	−0.008, 1.523	2.133
	**Service quality**	0.213	0.125	0.050	−0.033, 0.458	1.237
	**Drug treatment effect**					
	Common vs. bad	0.710	0.517	0.170	−0.304, 1.723	2.033
	Good vs. bad	1.108	0.538	0.039	0.053, 2.163	3.029
	**Drug price**					
	Suitable vs. low	0.011	0.298	0.971	−0.573, 0.595	1.011
	High vs. low	0.036	0.377	0.924	−0.704, 0.776	1.037
	Pseudo R^2^	0.094				
	Log likelihood	−343.094				
	Chi-squared	58.133				
	Akaike crit. (AIC)	694.144				
	Bayesian crit. (BIC)	732.998				
	*N*	574				
**Model 4**	**Sex**					
	Male vs. female	−0.383	0.200	0.055	−0.774, 0.008	0.682
	**Age**					
	41–59 vs. ≤40	−0.528	0.238	0.026	−0.994, −0.062	0.590
	≥60 vs. ≤40	−0.388	0.302	0.200	−0.980, 0.204	0.679
	**Education**					
	Middle school vs. primary school or lower	0.039	0.450	0.931	−0.843, 0.921	1.040
	High school or higher vs. primary school or lower	0.032	0.492	0.948	−0.932, 0.997	1.033
	**Income**					
	10,000–29,999 vs. ≤9999	−0.178	0.237	0.452	−0.642, 0.286	0.837
	≥30,000 vs. ≤9999	0.285	0.291	0.327	−0.285, 0.854	1.329
	**Reimbursement rate of NRCMI**					
	Suitable vs. low	−0.038	0.367	0.918	−0.756, 0.681	0.963
	High vs. low	0.758	0.391	0.052	−0.008, 1.524	2.134
	**Service quality**	0.006	0.138	0.964	−0.265, 0.277	1.006
	**Drug treatment effect**					
	Common vs. bad	0.847	0.519	0.103	−0.170, 1.863	2.332
	Good vs. bad	1.166	0.539	0.030	0.111, 2.222	3.211
	**Drug price**					
	Suitable vs. low	0.072	0.299	0.808	−0.513, 0.658	1.075
	High vs. low	0.166	0.382	0.664	−0.583, 0.915	1.180
	**Trust**	0.504	0.138	<0.001	0.233, 0.774	1.655
	Pseudo R^2^	0.112				
	Log likelihood	−336.092				
	Chi-squared	71.064				
	Akaike crit. (AIC)	710.183				
	Bayesian crit. (BIC)	792.850				
	*N*	574				
**Model 5**	High reimbursement	−1.279	0.749	0.088	−2.748, 0.189	0.278
	rate × trust					
	Drug treatment effect common × trust	2.151	1.132	0.057	−0.0682, 4.371	8.596
	Drug treatment effect					
	good × trust	2.158	1.150	0.061	−0.097, 4.412	8.655
	Pseudo R^2^			0.107		
	Log likelihood			−337.590		
	Chi-squared			92.051		
	Akaike crit. (AIC)			709.201		
	Bayesian crit. (BIC)			804.920		
	*N*			574		

β: regression coefficient; SE: standard error; OR: odds ratio; CI: confidence interval.

## Abbreviations

GP	General practitioner
NRCMI	New Rural Cooperative Medicine Insurance
ServQual	Service quality

## References

[1] Ding Y., Smith H.J., Fei Y., Xu B., Nie S., Yan W., Diwan V.K., Sauerborn R., Dong H. (2012). Factors influencing the provision of public health services by village doctors in Hubei and Jiangxi provinces, China. Bull. World Health Organ..

[2] Brant M.G.S. (2006). Edward Okeke and Josh Rosenfeld: Access to Care in Rural China: A Policy Discussion.

[3] Carrin A.R.G., Yang H., Wang H., Zhang T., Zhang L., Zhang S., Ye Y., Chen J., Jiang Q., Zhang Z. (1999). The reform of the rural cooperative medical system in the People’s Republic of China: Interim experience in 14 pilot countiesp. Soc. Sci. Med..

[4] Cao G.L., Zhang B. (1990). The Changes and the Development of the Cooperative Medical System in Our Country. Chin. Prim. Health Care.

[5] Liu X., Cao H. (1992). China’s Cooperative Medical System: Its Historical Transformations and the Trend of Development. Public Health Policy.

[6] Opinion on Reforming and Improving Training and Employment Incentive Mechanism of General Practitioner. http://www.gov.cn/zhengce/content/2018-01/24/content_5260073.htm.

[7] Zhou H., Zhang W., Zhang S., Wang F., Zhong Y., Gu L., Qu Z., Liang X., Sa Z., Wang X. (2015). Health providers’ perspectives on delivering public health services under the contract service policy in rural China: Evidence from Xinjian County. BMC Health Serv. Res..

[8] Zhou H., Zhang S., Zhang W., Wang F., Zhong Y., Gu L., Qu Z., Tian D. (2015). Evaluation and mechanism for outcomes exploration of providing public health care in contract service in rural China: A multiple-case study with complex adaptive systems design. BMC Public Health.

[9] Yuan S., Wang F., Li X., Jia M., Tian M. (2019). Facilitators and barriers to implement the family doctor contracting services in China: Findings from a qualitative study. BMJ Open.

[10] Ning W., Lina Y., Hao W., Zhang G. (2015). Research on progress, problems and strategies of rural doctors contract services. Chin. Health Econ..

[11] Fu P., Wang Y., Liu S., Li J., Gao Q., Zhou C., Meng Q., Sylvia S. (2020). Analysing the preferences for family doctor contract services in rural China: A study using a discrete choice experiment. BMC Fam. Pr..

[12] Chen A., Feng S., Tang W., Zhang L. (2019). Satisfaction with service coverage and drug list may influence patients’ acceptance of general practitioner contract service: A cross-sectional study in Guangdong, China. BMC Health Serv. Res..

[13] (2017). Statistical Bulletin of National Economic and Social Development of Dafeng District. http://www.dafeng.gov.cn/K1308267X/doc/2018/8/97872.shtml.

[14] (2018). Report on the Work of the Government of Yinan County. http://www.yinan.gov.cn/info/1078/12920.htm.

[15] (2018). Report on the Work of the Government of Wufeng. http://www.hbwf.gov.cn/content-365-464707-1.html.

[16] Gu L., Deng J., Xu H., Zhang S., Gao M., Qu Z., Zhang W., Tian D. (2019). The impact of contract service policy and doctor communication skills on rural patient-doctor trust relationship in the village clinics of three counties. BMC Health Serv. Res..

[17] Feng X. (2014). A study on urban-rural differences of gender role attitudes in Chinese women. J. Humanit..

[18] Feng X. (2013). Methods of Social Research.

[19] Shang X., Huang Y., Li B., Yang Q., Zhao Y., Wang W., Liu Y., Lin J., Hu C., Qiu Y. (2019). Residents’ Awareness of Family Doctor Contract Services, Status of Contract with a Family Doctor, and Contract Service Needs in Zhejiang Province, China: A Cross-Sectional Study. Int. J. Environ. Res. Public Health.

[20] Liu S., Yu C., Chen L. (2017). Assessment of service quality of family doctor service modelin rural areas based on ServQual. Anhui Med. J..

[21] Dong E., Bao Y. (2012). Reliability and validity of the Chinese version of Wake Forest Physician Trust Scale. Chin. Ment. Health J..

[22] Kang G.D., James J. (2004). Service quality dimensions: An examination of Gronroos’s service quality model. Manag. Serv. Qual..

[23] Alrubaiee L., Alkaa’ida F. (2011). The mediating effect of patient satisfaction in the patients’ perceptions of healthcare quality-patient trust relationship. Int. J. Mark. Stud..

[24] Liu Z., Tan Y., Liang H., Gu Y., Wang X., Hao Y., Gu J., Hao C. (2019). Factors influencing residents’ willingness to contract with general practitioners in Guangzhou, China, during the GP policy trial phase: A cross-sectional study based on Andersen’s behavioral model of health services use. Inq. J. Med. Care Organ. Provis. Financ..

[25] Wang H., Shi L., Han X., Zhang J., Ma Y., Yang X., Liu M., Fan L., Lou F. (2019). Factors associated with contracted services of Chinese family doctors from the perspective of medical staff and consumers: A cross-sectional study. BMC Health Serv. Res..

[26] Lina C., Zhong J., Mei Y., Liang K. (2018). Effect of family practice contract services on the quality of primary care in Guangzhou, China: A cross-sectional study using PCAT-AE. BMJ Open.

[27] Giddens A. (1990). The Consequences of Modernity.

[28] Plomp H.N., Ballast N. (2010). Trust and vulnerability in doctor-patient relations in occupational health. Occup. Med..

[29] Jones D.E., Carson K.A., Bleich S.N., Cooper L.A. (2012). Patient trust in physicians and adoption of lifestyle behaviors to control high blood pressure. Patient Educ. Couns..

[30] Farrington C. (2011). Reconciling managers, doctors, and patients: The role of clear communication. J. R. Soc. Med..

[31] Kangzhi Z. (2008). The interpersonal relationship between acquaintances and strangers. J. Jiangsu Adm. Inst..

[32] Kangzhi Z. (2005). See trust ih history coordinate. Soc. Sci. Res..

[33] Myint C.-Y., Pavlova M., Groot W. (2019). Catastrophic health care expenditure in Myanmar: Policy implications in leading progress towards universal health coverage. Int. J. Equity Health.

